# Dr. Thomas D. Spencer

**Published:** 1910-05

**Authors:** 


### OBITUARY

Dr. Thomas D. Spencer, of Rochester, formerly president of the medical staff of the Rochester Homeopathic Hospital, a graduate of the New York Homeopathic Medical College, 1878, died in the Homeopathic Hospital at Rochester, March 31, 1910, aged 53 years.

